# Thyroid Involvement in Hepatitis C Virus-Infected Patients with/without Mixed Cryoglobulinemia

**DOI:** 10.3389/fendo.2017.00159

**Published:** 2017-07-07

**Authors:** Clodoveo Ferri, Michele Colaci, Poupak Fallahi, Silvia Martina Ferrari, Alessandro Antonelli, Dilia Giuggioli

**Affiliations:** ^1^Chair and Rheumatology Unit, Azienda Ospedaliero-Universitaria di Modena, Medical School, University of Modena and Reggio Emilia, Modena, Italy; ^2^Department of Clinical and Experimental Medicine, University of Pisa, Pisa, Italy

**Keywords:** hepatitis C virus, thyroid, autoimmune thyroiditis, autoimmunity, cryoglobulinemia, cryoglobulinemic vasculitis, cancer, lymphoma

## Abstract

Thyroid involvement is a common condition that can be recorded during the natural course of different systemic rheumatic diseases, including the mixed cryoglobulinemia (MC) syndrome or cryoglobulinemic vasculitis. MC is triggered by hepatitis C virus (HCV) chronic infection in the majority of cases; it represents the prototype of autoimmune-lymphoproliferative disorders complicating a significant proportion of patients with chronic HCV infection. HCV is both hepato- and lymphotropic virus responsible for a great number of autoimmune/lymphoproliferative and/or neoplastic disorders. The complex of HCV-related hepatic and extrahepatic manifestations, including MC and thyroid involvement, may be termed “HCV syndrome.” Here, we describe the prevalence and clinico-serological characteristics of thyroid involvement, mainly autoimmune thyroiditis and papillary thyroid cancer, in patients with HCV syndrome with or without cryoglobulinemic vasculitis.

## Introduction

Autoimmune thyroiditis (AT) includes a group of thyroid diseases whose etiopathogenesis is characterized by chronic inflammatory response specifically self-directed against thyroid gland (1–3). Hashimoto’s thyroiditis and Graves’ disease represent the main pathophysiological and clinical entities of this single organ autoimmune disorder; nonetheless, subclinical thyroid dysfunctions should be considered in the disease spectrum (1, 2). Clinically, AT can lead to both hyper- and hypothyroidism, more often the latter, or it can produce slight, insidious variations of the TSH levels, without overt manifestations (1, 2). Presence of AT in the course of autoimmune systemic diseases, including mixed cryoglobulinemia (MC), is very frequent.

**Figure 1:**
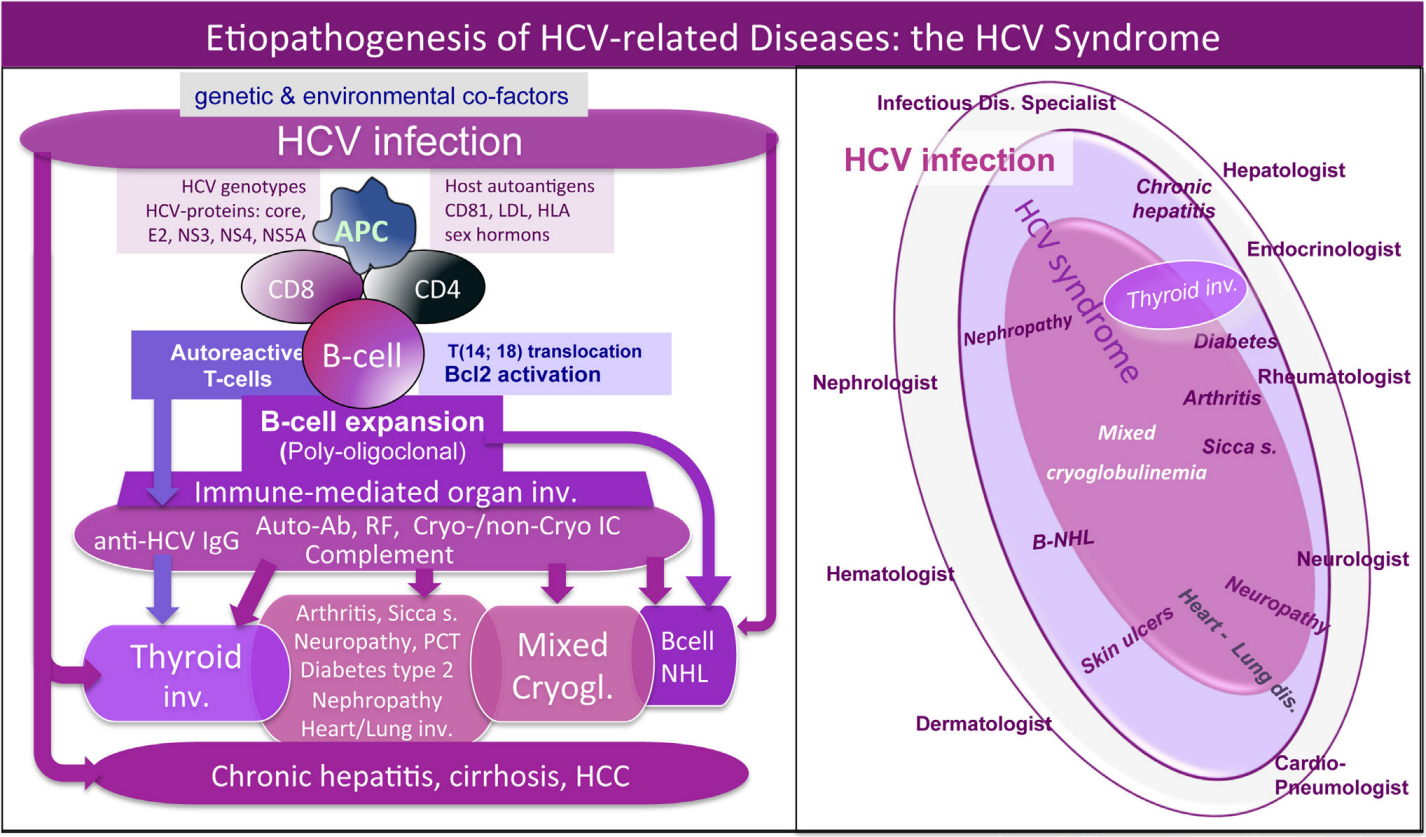
Etiopathogenesis of hepatitis C virus (HCV)-related disorders and HCV syndrome. *Left*: the etiopathogenesis of HCV syndrome includes both hepatic and extrahepatic disorders. They may develop through a multifactorial and multistep process that includes chronic HCV infection, other potential environmental/toxic triggers, genetically driven host predisposition (particularly HLA alleles, metabolic, and/or hormonal factors), and complex cellular and molecular alterations. From one side, we can observe HCV-driven immune-system alterations with prominent “benign” lymphoproliferation and autoantibody production, from the other side, deeper oncogenetic alterations leading to frank B-cell neoplasias and other malignancies (B-NHL, HCC, and papillary thyroid cancer). These different pathogenetic mechanisms are not mutually exclusive; during long-term follow-up, we can assist in the same HCV-infected patient to the appearance of different organ- and non-organ-specific autoimmune/neoplastic diseases, among which thyroid involvement. HCV antigens (core, envelope E2, NS3, NS4, NS5A proteins) may exert a chronic stimulus on the host immune system. Important pathogenetic steps include high-affinity binding between HCV-E2 and CD81 and consequent *t*(14;18) translocation with bcl-2 proto-oncogene activation, cross-reaction between particular HCV antigens and host autoantigens (molecular mimicry mechanism), and direct cell infection by HCV responsible for neoplastic cell transformation. The “benign,” often subclinical, B-cell proliferation with production of various autoantibodies, among which RF and cryo- and non-cryoprecipitable immune complexes may be frequently observed in chronically HCV-infected individuals. This condition may be the pathological substrate of various organ- and non-organ-specific autoimmune disorders, including thyroid involvement with/without mixed cryoglobulinemia (MC) syndrome or cryoglobulinemic vasculitis. Complicating malignancies can be observed in a small but significant percentage of patients, usually as a late complication of chronic HCV infection; moreover, both autoimmune and neoplastic disorders show a clinico-serological and pathological overlap. *Right*: schematic reproduction of the so-called “HCV syndrome” that encompasses the variety of HCV-related diseases. The majority of HCV-infected patients may remain totally asymptomatic or complicated by isolated liver involvement; however, a significant proportion of subjects may develop various extrahepatic manifestations that may include a variety of autoimmune/lymphoproliferative and neoplastic disorders; therefore HCV-positive patients are commonly referred to different specialists according the prevalent clinical manifestation(s). A number of HCV-infected patients may be referred early to the rheumatologist because of mild clinical manifestations such as arthralgias/mialgias, sicca syndrome, and/or RF seropositivity. MC syndrome, also termed cryoglobulinemic vasculitis, represents the prototype of extrahepatic, immune-mediated systemic disorder characterized by multiple organ involvement. In this scenario, HCV-related thyroid involvement is one of the most frequent manifestations, isolated or in association with other extrahepatic disorders, mainly cryoglobulinemic vasculitis. RF, rheumatoid factor; NHL, non-Hodgkin’s lymphoma; HCC, hepatocellular carcinoma; PCT, porphyria cutanea tarda.

Mixed cryoglobulinemia is a small-vessel vasculitis due to vessel deposition of cryo- and non-cryoprecipitable IgG–IgM immune complexes (ICs) and complement, which represent the main pathogenetic mechanism of disease manifestations, such as palpable purpura of the legs, skin ulcers, peripheral polyneuropathy, or glomerulonephritis; moreover, arthralgias, fatigue, sicca syndrome are frequently associated (4–7). The abnormal production of ICs is determined by B cell clone proliferation triggered by hepatitis C virus (HCV) in a small proportion of infected patients (Figure 1, left). Besides its well-known hepatic tropism, HCV is able to infect several cell types (including B cells and thyrocytes); therefore, HCV lymphotropism can stimulate autoimmunity due to benign B-lymphocyte expansion or malignant B-cell non-Hodgkin’s lymphoma [(5–7); Figure 1, left]. Therefore, HCV patients frequently present a variable combination of different organ and/or systemic autoimmune diseases and neoplasias. The proposed “HCV syndrome” encompasses the complex of both hepatic and extrahepatic disorders [(7); Figure 1, right] among which the MC, also called cryoglobulinemic vasculitis, is the pathophysiological and clinical prototype (8).

As deeply described below, AT may be frequently found in HCV patients, with or without MC syndrome, suggesting an etiopathogenetic role of the virus in a subset of predisposed subjects (9–12).

## Thyroid Disease Associated with HCV

Thyroid involvement is considered one of the most frequent endocrine disorders in association with chronic HCV infection, independently from the presence of MC (13); in particular, AT may represent a frequent extrahepatic disease in the spectrum of HCV syndrome (4, 7, 13).

A large Italian population-based study published in 2004 (14) investigated the prevalence of AT (including thyroid dysfunction) in a series of 630 HCV patients not treated with interferon (IFN)-alpha, compared with three control groups: 389 individuals from an iodine-deficient area, 268 individuals from an area of iodine sufficiency, and 86 patients with chronic hepatitis B. HCV patients were more likely to have hypothyroidism (13%), anti-thyroglobulin antibodies (TgAb) (17%), and anti-thyroperoxidase antibodies (TPOAb) (21%) than the control groups (14).

A retrospective cohort study (15) analyzing data of users of US Veterans Affairs health-care facilities from 1997 to 2004 (146,394 HCV-infected patients vs. 572,293 HCV negatives) found that thyroiditis risk was slightly increased (adjusted hazard ratio 1.13, 95% CI 1.08–1.18; *p* < 0.001). It is supposable that the increased AT prevalence was underestimated because of 97% of cases were males, considering that both AT and hypothyroidism are associated with the female gender (12).

Other studies investigating the frequency of AT in smaller HCV patient cohorts were analyzed by Shen et al. in a recent meta-analysis of the world literature on the topic (16). Totally, 1,735 HCV-infected and 1,868 non-HCV subjects were pooled; prevalence of TgAb, TPOAb, and anti-thyroid microsomal antibody were 2.40-, 1.96- and 1.86-fold higher in HCV-positive subjects than in controls. Moreover, the hypothyroidism risk is 3.10 (95% CI 2.19–4.40) in HCV-infected patients.

Up to the recent introduction of new antivirals, the mainstay of HCV treatment was the IFN-alpha in combination with ribavirin. Several HCV patients developed AT as a consequence of IFN therapy, possibly due to the stimulation of antithyroid antibodies production in predisposed subjects (17). Frequently, IFN-related AT resolves within a few months (9).

## Thyroid Disease Associated with MC

The coexistence of AT and MC has been reported in large cohort studies evaluating the clinico-serological characteristics of HCV-infected patients (15, 18). However, a definite association between these two diseases was first investigated by an Italian case–control prospective study (19), including 93 patients affected by HCV-related MC, 93 patients with isolated type C hepatitis, and 93 age/sex-matched healthy subjects from the same geographical area as controls. AT, subclinical hypothyroidism, and the presence of isolated specific serum autoantibodies, i.e., TPOAb and/or TgAb, were more frequent in the first group than the controls (35 vs. 16%, 11 vs. 2%, 31 vs. 12%, respectively). Moreover, higher frequency of AT was recorded among MC patients in comparison with hepatopathic patients (35 vs. 22%), with a significant high prevalence of TPOAb in MC (28 vs. 14%). Finally, hypothyroidism was associated with higher cryocrit and with the presence of other autoantibody positivity, as well as with longer MC duration, presence of proteinuria, or active hepatitis (19).

These findings showed that AT patients exhibited more pronounced autoimmune phenomena and severity of MC, which represents the prototype of autoimmunity in HCV patients (7).

Moreover, a longitudinal study investigating the incidence of AT during the follow-up of 112 MC patients vs. 112 matched controls was recently carried out (20). Of interest, the appearance of new cases of AT were evidenced during the course of HCV infection besides the observed AT at baseline; in particular, after a median of 67 and 96 months of follow-up in the two groups of HCV-positive patients with or without MC, AT was newly reported in 14 MC patients and in three controls; consequently, the overall prevalence of AT was increased up to 33 and 16%, respectively. Moreover, hypothyroidism that was invariably absent at baseline developed in 11 MC patients and three controls (subclinical in 9/11 vs. 2/3, respectively), while no cases of Grave’s disease were registered. Interestingly, the logistic regression analysis confirmed that the appearance of hypothyroidism was related to female gender, a well-known risk factor for autoimmunity.

Noteworthy, even the prevalence of papillary thyroid cancer resulted higher in MC compared to hepatitis C patients; namely, the same study found two cases of cancer among MC patients, but none in hepatitis C and healthy controls (19). Subsequently, thyroid cancer was first reported also in patients with HCV infection regardless of the presence of other HCV-related manifestations (21); this finding supported the hypothesis of the oncogenic potential of HCV through the direct infection of thyrocytes with the possible contribution of the pathogenetic process responsible for AT (22, 23). Essentially, this latter reproduces the same multistep process already demonstrated for HCV lymphotropism with “benign” B-cell proliferation and subsequent lymphomagenesis (6, 7).

## Pathogenesis

Clinico-epidemiological studies largely demonstrated that chronic HCV infection is a relevant risk factor for the development of a number of autoimmune or neoplastic diseases, including thyroid involvement, mainly AT (4, 5, 7–10). Considering this latter manifestation, an important contribute to understand the mechanisms involved in the pathogenesis of thyroid disorders was given by Blackard et al. (24), who demonstrated that HCV may infect a human thyroid cell line (ML1), which presents the membrane expression of the important HCV receptor CD81.

**Figure 2:**
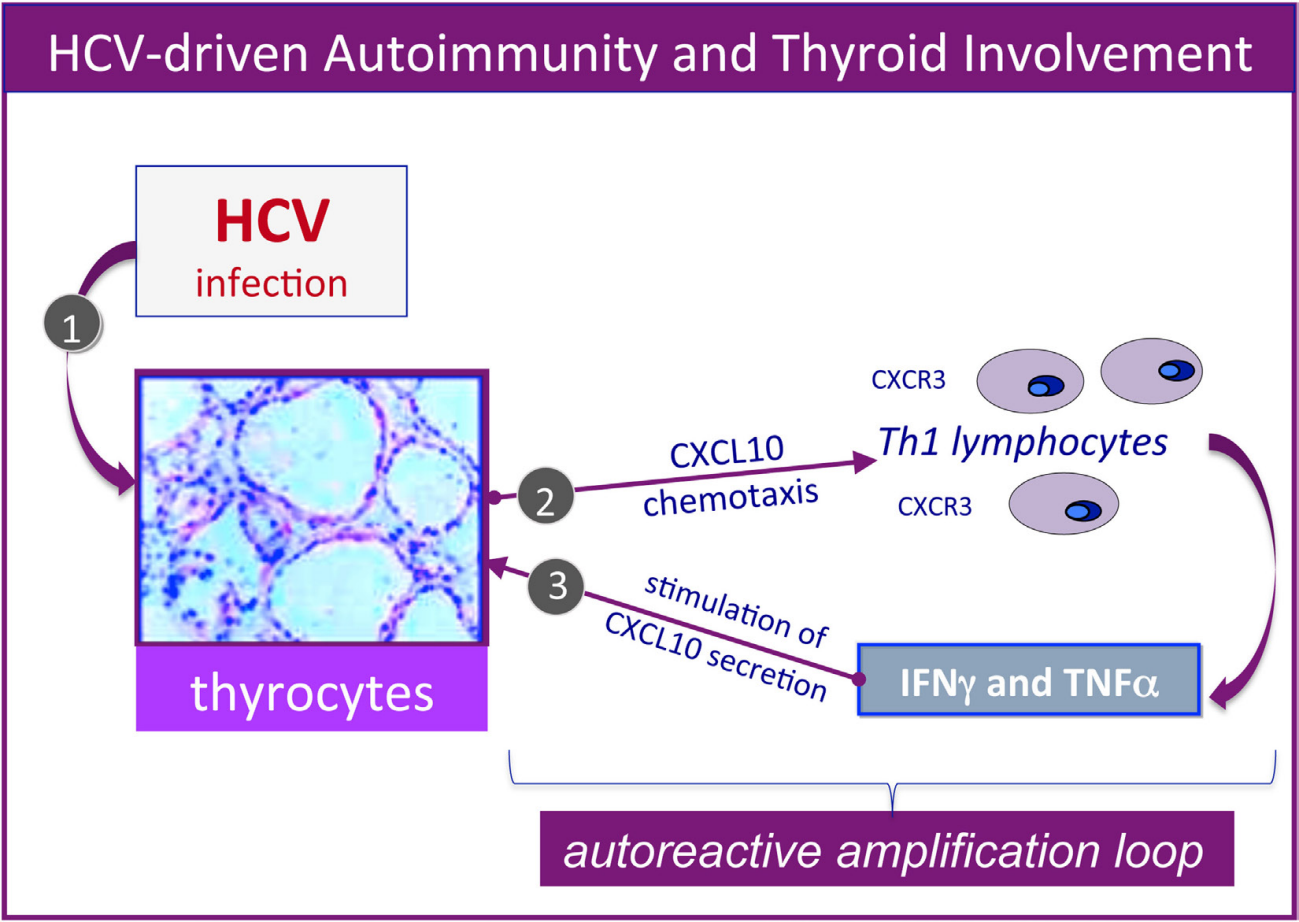
Hepatitis C virus (HCV)-driven autoimmunity and thyroid involvement. Autoimmune thyroid involvement can be observed in a significant proportion of chronically HCV-infected patients; the possible etiopathogenetic mechanisms are schematically described in the figure. In genetically predisposed subjects, HCV thyroid infection (1) may lead to the upregulation of CXCL10 gene expression and secretion in thyrocytes (2); this chemokine may promote the recruitment of Th1 lymphocytes, which secrete interferon-γ (IFNγ) and tumor necrosis factor-α (TNFα). These cytokines may in turn induce CXCL10 secretion by thyrocytes (3), thus perpetuating the immune-mediated pathogenetic cascade. The consequence may be the appearance of thyroid disorders; a comparable pathogenetic mechanism may be hypothesized for HCV-driven diabetes type 2.

Furthermore, several studies by our group reported the upregulation of the CXCL9, CXCL10, CXCL11 chemokines, as well as IL-6 in the serum of MC patients who also presented AT (25–29). Therefore, it could be hypothesized that HCV may lead toward chronic stimulation of the immune system (Figure 1, left), namely the T-helper 1 lymphocytes, which secrete interferon-gamma and tumor necrosis factor-alpha, that in turn perpetuate the immune cascade increasing the levels of the chemokines cited above (Figure 2). Finally, the sustained activation of the immune system is at the basis of thyroid immune-mediated damage, leading to AT and other important disorders such as papillary thyroid cancer (21, 22).

## Conclusion

Autoimmune thyroiditis diagnosis is relatively simple and is based on typical laboratory and instrumental findings. The main autoantibodies of AT are the TPOAb and TgAb, while those directed against the TSH receptor are typical of Graves disease (1–3, 8). Histologically, lymphocytes infiltrate the thyroid parenchyma, even forming lymphoid follicles, progressively leading to parenchymal destruction and glandular fibrosis (3); anyway, thyroid biopsy is generally not required for the diagnosis. Instead, ultrasounds are usually important to support the AT diagnosis, identifying heterogeneous pattern of the gland, up to pseudo-nodular feature (1–3). The instrumental follow-up is obviously important to precociously diagnose the cases of thyroid cancer.

Considering the relative feasibility of AT diagnosis using not expensive or invasive exams, all HCV patients, mainly if affected by MC, should undergo thyroid evaluation periodically.

In the majority of MC patients, AT is a silent part of the clinical picture; otherwise, hypothyroidism, more frequently subclinical, may develop (9, 19, 20). The standard hormone replacement therapy is indicated in symptomatic HCV-associated AT with/without MC syndrome. HCV eradication is an important therapeutical/preemptive approach to several manifestations of HCV syndrome (30), including thyroid involvement, in particular the rare HCV-related papillary thyroid cancer.

## Author Contributions

CF: general revision and images drawing. MC: literature review and article writing. PF: literature revision. SF: literature revision. AA: general revision as regards endocrinology. DG: literature review and article writing.

## Conflict of Interest Statement

All the authors declare the absence of any relationship with a commercial company that has a direct financial interest in subject matter or materials discussed in article or with a company making a competing product. The reviewer, SM, and handling editor declared their shared affiliation, and the handling editor states that the process nevertheless met the standards of a fair and objective review.
